# The Structural Basis of Antibody-Antigen Recognition

**DOI:** 10.3389/fimmu.2013.00302

**Published:** 2013-10-08

**Authors:** Inbal Sela-Culang, Vered Kunik, Yanay Ofran

**Affiliations:** ^1^The Goodman Faculty of Life Sciences, Bar Ilan University, Ramat Gan, Israel

**Keywords:** antibody, CDRs, antigen, paratope, epitope, framework, constant domain

## Abstract

The function of antibodies (Abs) involves specific binding to antigens (Ags) and activation of other components of the immune system to fight pathogens. The six hypervariable loops within the variable domains of Abs, commonly termed complementarity determining regions (CDRs), are widely assumed to be responsible for Ag recognition, while the constant domains are believed to mediate effector activation. Recent studies and analyses of the growing number of available Ab structures, indicate that this clear functional separation between the two regions may be an oversimplification. Some positions within the CDRs have been shown to never participate in Ag binding and some off-CDRs residues often contribute critically to the interaction with the Ag. Moreover, there is now growing evidence for non-local and even allosteric effects in Ab-Ag interaction in which Ag binding affects the constant region and vice versa. This review summarizes and discusses the structural basis of Ag recognition, elaborating on the contribution of different structural determinants of the Ab to Ag binding and recognition. We discuss the CDRs, the different approaches for their identification and their relationship to the Ag interface. We also review what is currently known about the contribution of non-CDRs regions to Ag recognition, namely the framework regions (FRs) and the constant domains. The suggested mechanisms by which these regions contribute to Ag binding are discussed. On the Ag side of the interaction, we discuss attempts to predict B-cell epitopes and the suggested idea to incorporate Ab information into B-cell epitope prediction schemes. Beyond improving the understanding of immunity, characterization of the functional role of different parts of the Ab molecule may help in Ab engineering, design of CDR-derived peptides, and epitope prediction.

## Introduction

Antibodies (Abs) have two distinct functions: one is to bind specifically to their target antigens (Ags); the other is to elicit an immune response against the bound Ag by recruiting other cells and molecules. The association between an Ab and an Ag involves myriad of non-covalent interactions between the epitope – the binding site on the Ag, and the paratopes – the binding site on the Ab. The ability of Abs to bind virtually any non-self surface with exquisite specificity and high affinity is not only the key to immunity but has also made Abs an enormously valuable tool in experimental biology, biomedical research, diagnostics and therapy. The diversity of their binding capabilities is particularly striking given the high structural similarity between all Abs. The availability of increasing amounts of structural data in recent years now allows for a much better understanding of the structural basis of Ab function in general, and of Ag recognition in particular. This review surveys the recent developments and the current gaps and challenges in this field. We focus specifically on the current understanding of the determinants within the Ab structure that contribute to Ag binding. We first discuss the motivations for, and applications of, the study of the structural basis of Ag recognition. Then we describe and discuss the Ab-Ag interface, with specific focus on the paratopes and the complementarity determining regions (CDRs), and their role in Ag binding. The last part focuses on the contribution of the non-CDRs parts of the Ab [i.e., framework regions (FRs) and the constant domains] to Ag binding and on the recent suggestions regarding non-local and allosteric effects in Ab function. Over the last few years numerous reviews have addressed issues that are related or tangential to the topics we review here. This includes reviews of the engineering of Abs (1), their stability (2), affinity maturation (3), and isotype selection (4). While these important topics are relevant to the findings and ideas we review here, they are beyond the scope of this review.

## The Motivations for, and Applications of, the Study of Ab-Ag Recognition

### Understanding immunity and autoimmunity

The adaptive immune response involves two types of lymphocytes: T cells, which recognizes Ags that have been processed and their fragments are presented by MHC molecules, and B cells which produce soluble Abs that can identify also the intact Ag in its native form. While the way in which T cells recognize their epitopes has been extensively studied to a level that enables the successful prediction of T-cell epitopes (5, 6), the rules that govern Ab-Ag recognition, including which parts of the Ab structure underlie Ag recognition and how and why certain determinants on the Ag are selected as epitopes, are not as well characterized. Understanding the mechanisms that underlie Ab-Ag recognition, therefore, is crucial for understanding immunity.

The immune system enables Abs to distinguish between foreign and self molecules (7). Autoimmune diseases are characterized by the inappropriate response to self-Ags. It is not always clear what role is played by Abs and what role is played by other components of the immune system in autoimmunity. A variety of molecular mechanisms have been proposed, including sequestered Ags, molecular mimicry, and polyclonal B-cell activation (8). Better understanding of the underpinnings of Ab-Ag recognition may also shed light on these questions.

### A model for studying bio-molecular recognition

A fundamental characteristic of the immune system is its ability to continuously generate novel protein recognition sites. Ab-Ag interfaces, therefore, are often considered a model system for elucidating the principles governing biomolecular recognition (9–13). For example, Keskin (14) and McCoy et al. (15) used X-ray crystallographic structures of Ab-Ag complexes to elucidate principles of the molecular architecture of protein–protein interfaces. Other studies, however, view Ab-Ag interfaces as a specific case that may not allow for generalization to all types of protein–protein interfaces (16). Thus, large scale studies of protein–protein interactions often exclude Ab-Ag complexes from the dataset analyzed (16–19). It is, therefore, important to determine to what extent Ab-Ag complexes could serve as a general model for protein–protein interactions.

### Antibody engineering

The specificity of the Ab molecule to its cognate Ag has been exploited for the development of a variety of immunoassays, vaccinations, and therapeutics. Ab engineering may offer to expand the application of Abs by permitting improvements of affinity (20, 21) and specificity (22, 23). Understanding of the role each structural element in the Ab plays in Ag recognition is essential for successful engineering of better binders. The engineering of Abs is also important for the clinical use of Abs from non-human sources. Early studies on the use of rodent Abs in humans determined that they can be immunogenic (24). Humanization by grafting of the CDRs from a mouse Ab to a human FR is a commonly used engineering strategy for reducing immunogenicity (25, 26). In most cases, the successful design of high-affinity, CDR-grafted, Abs requires that key residues in the human acceptor FRs that are crucial for preserving the functional conformation of the CDRs will be back-mutated to the amino acids of the original murine Ab (26, 27). Several groups (28–30) used the experimentally determined 3-D structures of Ab-Ag complexes in the Protein Data Bank (PDB) (31) to determine which residues participate in Ag recognition and binding. Such knowledge can be exploited to identify residues that are important for the function of the Ab in general and for Ag recognition in particular and may guide Ab engineering (32, 33). Residues that help maintain the functional conformation of the CDRs, for example, can be used to improve Ab humanization efforts by CDR-grafting.

### Ab epitope prediction

**Figure 1:**
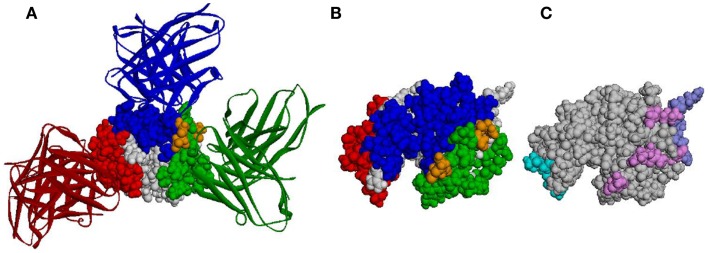
**Predicted epitopes vs. the actual epitopes of HEL**. **(A)** The 3-D structure of HEL (CPK representation) together with three Abs (ribbon representation). PDB IDs 1JHL, 3D9A, and 1MLC were superimposed according to HEL structure. Epitope residues are colored blue, green, and red according to the corresponding Ab. Residues that are common to two epitopes are colored orange. **(B)** The structure of HEL colored according to the same three epitopes as in **(A)**, presented in a different orientation. **(C)** The structure of HEL colored according to the epitopes predicted by Discotope (light blue), ellipro (purple), and seppa (pink). Note, not all predicted residues of Discotope and ellipro are observable in the presented orientation.

Antibody epitopes (sometimes referred to as B-cell epitopes) are the molecular structures within an Ag that make specific contacts with the Ab paratope. B-cell epitopes are used in the development of vaccines and in immunodiagnostics. Correct identification of B-cell epitopes within an antigenic protein, may open the door for the design of molecules (biologic or synthetic) that mimic potentially protective epitopes and could be used to raise specific Abs or be used as a prophylactic or therapeutic vaccines. Identification of B-cell epitopes could promote protective immunity in the context of emerging and re-emerging infectious diseases and potential bioterrorist threats. This may be achieved by choosing from among the putative epitopes those that may provide immunity (e.g., by eliciting Abs that hamper the molecular function of pathogenic Ags). The choice of such epitopes is believed to be relevant for understanding and controlling protective immunity. In the case of the vaccinia virus, for example, which was used as smallpox vaccine and is the only vaccine that has led to the complete eradication of an infectious disease from the human population, individuals possessing a high frequency of memory B-cells specific for major neutralizing Ags of the vaccinia virus are better protected from smallpox than individuals with a memory B-cell pool dominated by specificities for non-protective Ags (34). Thus, understanding the way in which an Ab recognizes its cognate epitope is of particular interest for vaccine design and disease prevention (35). Existing tools for identification of Ab epitopes (such as X-ray crystallography, pepscan, phage display, expressed fragments, partial proteolysis, mass spectrometry, and mutagenesis analysis) are not only expensive, laborious, and time consuming but also fail to identify many epitopes (36). When talking about protein Ags, most of these methods typically identify linear stretches as epitopes, while, arguably, most of the epitopes on protein Ags are conformational and even discontinuous. As for computational approaches, despite more than 30 years of efforts (37), existing B-cell epitope prediction methods are not accurate enough (38, 39) and are, therefore, not widely used. This is exemplified in Figure 1, in which the structure of hen egg lysozyme (HEL) Ag and three Abs that bind it are shown (Figures 1A,B), as well as the epitopes predicted by three different methods (Figure 1C).

In general, current methods are trying to identify epitopic residues based on the presence of features associated with residues that bind the Ab (40–50). One possible explanation for the failure of these methods is that the differences between epitopes and other residues are not substantial. Indeed, several analyses (51–53) have shown that the amino-acid composition of epitopes is essentially indistinguishable from that of other surface-exposed non-epitopic residues.

This lack of intrinsic properties that clearly differentiate between epitopic and non-epitopic residues and the fact (demonstrated in Figure 1) that most of the Ag surface may become a part of an epitope under some circumstances (54–57) suggest that epitopes depend, to a great extent, on the Abs that recognize them. This is exemplified in Figure 1: most of the HEL surface residues are part of an epitope of at least one Ab (Figures 1A,B), even though this figure shows only three Abs (out of dozens known to bind HEL). Almost all the residues predicted to be epitopic may be considered as correct predictions as they bind some Ab (Figure 1C) but also as false predictions as they don’t bind the others. Similarly, predicting that a residue is not in an epitope may be either a true negative or a false negative, depending on the Ab considered. It has recently been suggested by us (Sela-Culang et al., submitted) and by others (58–60) that predicting epitopes should be done for a certain Ab. A similar concept was successfully applied in the case of T-cell epitope prediction methods: these methods do not examine the Ag for general features. Rather, different predictions are made, dependent on the specific MHC molecule binding and presenting the epitope to T cells.

## The Role of CDRs and Their Definition

**Figure 2:**
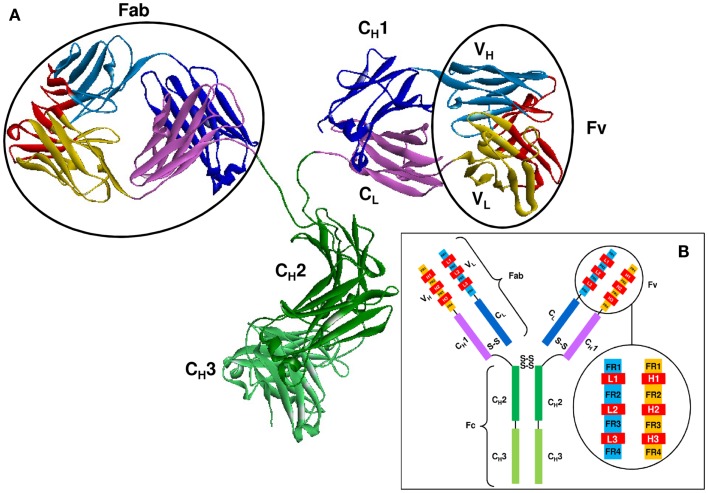
**The structure of an Ab molecule**. **(A)** The 3-D structure of an Ab molecule (PDB ID: 1IGT). **(B)** A schematic representation of the Ab scaffold.

As shown in Figure 2, Abs are all-beta proteins consisting of four polypeptide chains: two identical heavy (H) chains and two identical light (L) chains (61). The light and heavy chains are linked by disulfide bonds to form the arms of a Y-shaped structure, each arm is known as a Fab (61). The Fab is composed of two variable domains (VH in the heavy chain and VL in the light chain) and two constant domains (CH1 and CL) (62). In the pairing of light and heavy chains, the two variable domains dimerize to form the Fv fragment which contains the Ag binding site. Within each variable domain lie six hypervariable loops (63), three in the light chain (L1, L2, and L3) and three in the heavy chain (H1, H2, and H3), supported by a conserved FR of β-sheets. The light and heavy variable domains fold in a manner that brings the hypervariable loops together to create the Ag binding site or paratope. Two additional domains of the heavy chain, CH2, and CH3, compose the Fc region which is responsible for mediating the biological activity of the Ab molecule.

### CDRs identification

As indicated by their names, CDRs are believed to account for the recognition of the Ag. Therefore, a major focus in analyzing the structural basis for Ag recognition has been in identifying the exact boundaries of the CDRs in a given Ab. It is a common practice to identify paratopes through the identification of CDRs. Kabat and co-authors (63, 64) were the first to introduce a systematic approach to identify CDRs in newly sequenced Abs. It was based on the assumption that CDRs are the most variable regions between Abs. Therefore, they aligned the (fairly limited) set of Ab sequences available at that time and identified the most variable positions. Based on the alignment, they introduced a numbering scheme for the residues in the hypervariable regions and determined which positions mark the beginning and the end of each CDR. As structural data became available, Chothia and Lesk (65, 66) manually analyzed a small number of experimentally solved 3-D structures and determined the structural location of the loop regions. The boundaries of the FRs and CDRs were determined and the latter have been shown to adopt a restricted set of conformations, based on the presence of certain residues at key positions in the CDRs and the flanking FRs. Their finding that Kabat’s definitions of L1 and H1 are structurally incorrect led to the introduction of the Chothia numbering scheme. With the increase of available structural data, they ran their analysis anew and introduced a new definition of L1 (66) in 1989. In 1997 (67), however, they concluded that this correction was erroneous, and reverted to their original 1987 numbering scheme. While the Kabat and Chothia schemes treated separately the different families of immunoglobulin domains, Lefranc and colleagues (68, 69) proposed a unified numbering scheme (referred to as IMGT numbering scheme) for immunoglobulin variable domain genomic sequences, including Ab light and heavy variable domains, as well as T-cell receptor variable domains. To correlate between the sequence, structure, and domain folding behavior of all immunoglobulin variable domains, the Aho numbering scheme spatially aligned known 3-D structures of immunoglobulins and unified their numbering (70).

A drawback of the Kabat, Chothia, and IMGT numbering schemes is that CDRs length variability takes into account only the most common loop lengths; While both Kabat and Chothia schemes accommodate insertions with insertion letters (e.g., 27A), the IMGT scheme avoids the use of insertion codes for all but the least common very long loops, and the Aho numbering scheme places insertions and deletions symmetrically around a key position. However, Abs with unusually long insertions may be hard to annotate using these methods and, as a result, their CDRs may not be identified correctly. For instance, the recently determined 3-D crystal structure of two bovine Abs (71) reveal exceptionally long H3 CDRs ( >60 residues), with long insertions which these methods cannot accommodate and thus cannot identify the CDRs of these Abs.

### Are CDRs good proxies for the paratope?

While identification of paratopes is often done through identification of CDRs, not all the residues within the CDRs bind the Ag. In fact, an early analysis of the 3-D structures of Abs suggested that only 20–33% of the residues within the CDRs participate in Ag binding (72). In 1996, MacCallum and colleagues (73) performed a detailed residue-level analysis of Ag contacts. They suggested that contacting residues are more common at CDRs residues which are located at the center of the Ag combining site, and that non-contacting residues within the CDRs correspond with residues that are important for maintaining the structural conformations of the hypervariable loops and not necessarily for recognition of the Ag. Thus, they introduced a mapping of Ag-contacting propensities for each Ab position and proposed a new definition for CDRs based on these propensities. Padlan and co-workers (28) utilized Abs sequence and structure data to perform a by-position summary of Ag contacts. They found that the residues that are directly involved in the interaction with the Ag are also, in general, the most variable ones. They suggested that the residues that interact with the Ag should be called Specificity Determining Residues (SDRs).

**Figure 3:**
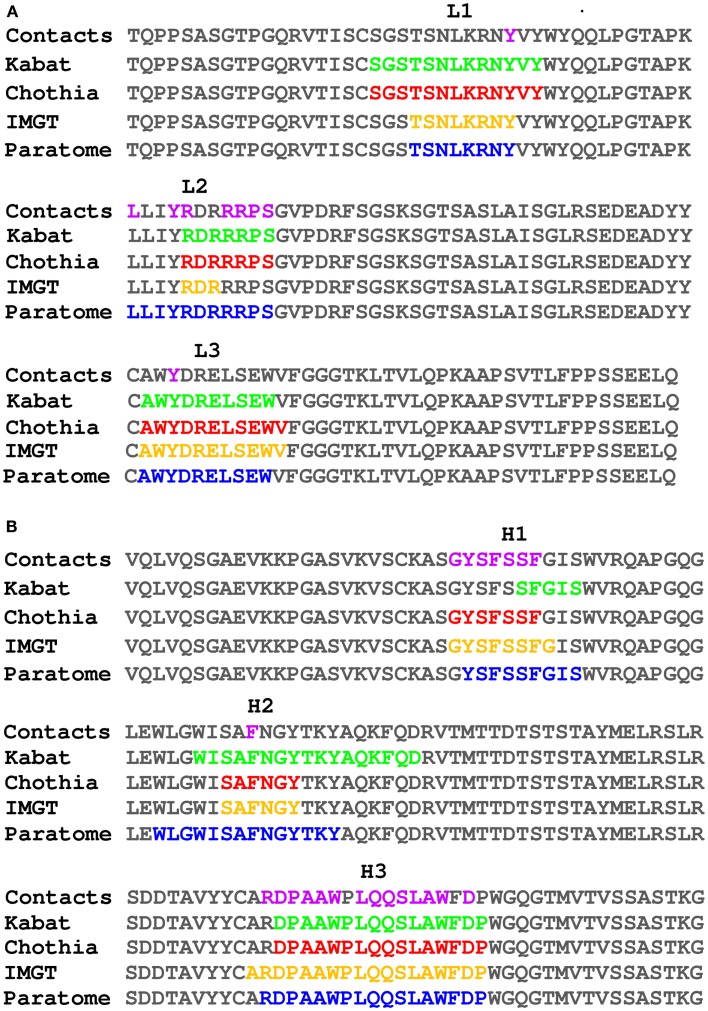
**Comparison of different CDR identification methods**. The light **(A)** and heavy **(B)** chains of PDB ID 2XQB were numbered according to Kabat (colored green) and Chothia (colored red) using the Abnum tool (www.bioinf.org.uk/abs/abnum) and CDRs were extracted according to the CDR definitions table (www.bioinf.org.uk/abs/#cdrs). CDRs according to IMGT (colored orange) were identified using the IMGT-gap tool (www.imgt.org/3Dstructure-DB/cgi/DomainGapAlign.cgi). ABRs according to Paratome (colored blue) were identified using the Paratome server (www.ofranlab.org/paratome). Contacts (colored purple) between the Ab and IL-15 were defined using a 6-Å cutoff value.

**Figure 4:**
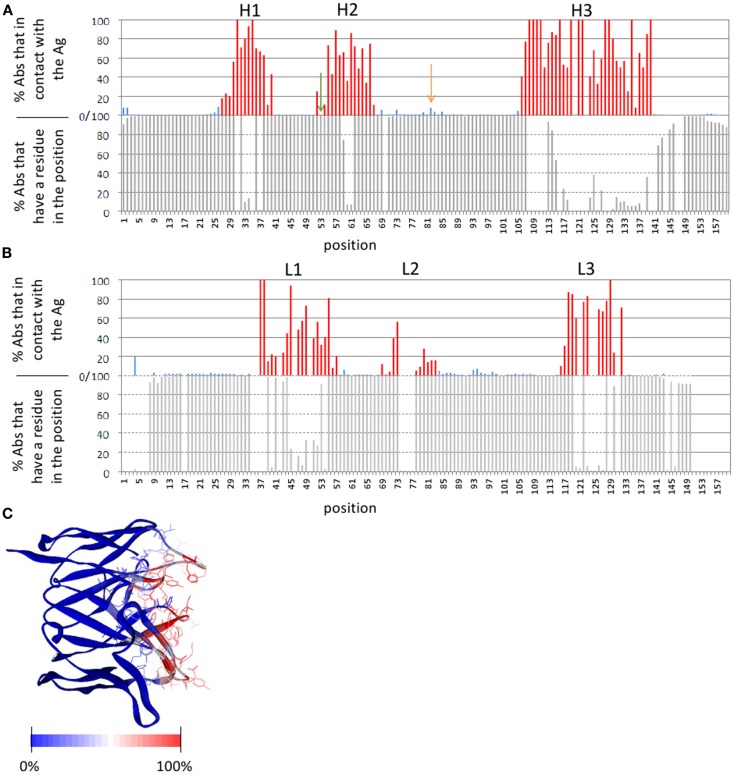
**Ab positions that contact the Ag**. **(A,B)** The lower graphs show the percentage of Abs with known 3-D structure that have a residue in a given position (i.e., in other Abs there is a gap in the MSTA in that position). The upper graphs show the percentage of Abs that contact the Ag out of those Abs that have a residue in that position. **(A)** Depicts the heavy chain and **(B)** depicts the light chain. In the upper graphs, the ABRs are colored red and the FRs are colored blue. An example of a position within an ABR that is not in contact with the Ag in any of the Abs, is marked by a green arrow. An example of a position in the FRs that is in contact with the Ag in many (8%) of the Abs is marked by an orange arrow. **(C)** The Ab Fv domain (PDB ID: 1QFU) is colored according to the percentage of all Abs with known 3-D structure in which the residue in that position is in contact with the Ag: from red (100% of the Abs) to blue (0%). ABR residues are presented as lines. The definition of the ABRs is according to the Paratome server. A 6-Å cutoff value was used to define residues in contact. Percentages of contacts were calculated based on an MSTA of all protein Ab-Ag complexes in the PDB (30).

The number of publicly available structures of Ab-Ag complexes increased in recent years to a level that enabled large-scale analyses. In a recent analysis (29) we utilized all available protein-Ab complexes in the PDB to identify the structural regions in which Ag binding actually occurs. This approach was implemented into a method dubbed Paratome (30, 74) that is based on a multiple structure alignment (MSTA) of all available Ab-Ag complexes in the PDB. The MSTA revealed regions of structural consensus where the pattern of structural positions that bind the Ag is highly similar among all Abs. These regions of structural binding consensus were termed antigen binding regions (ABRs). While CDRs, as identified by methods such as Kabat (63), Chothia (65), and IMGT (69), may miss ∼20% of the Ag binding residues, ABRs cover ∼96% of the residues that actually bind the Ag (30). To avoid confusions and cumbersome nomenclature, herein we generically refer to CDRs, SDRs, and ABRs as “CDRs” unless otherwise specified. Figure 3 shows an example of CDRs as identified by Kabat, Chothia, IMGT, and Paratome for one Ab (anti-IL-15, PDB ID: 2XQB), compared to the actual Ag binding residues. It can be seen that in this example, some of the CDRs (e.g., L3, H3) identified by the four methods are almost identical, while in other CDRs (e.g., L2, H1, and H2) there are substantial differences between the methods. The MSTA of Abs with known 3-D structure also confirmed previous observations that there are structural positions within the CDRs in which none, or only a small percentage of the Abs contact the Ag. This is shown in Figure 4 where an example of such a position is marked by a green arrow.

### Integrality vs. modularity

Designed systems are often characterized as either modular or integral. In a modular system different components, or modules, function independent of the function of other modules. The generation of Abs in the immune system is based on combining different elements, in a way that may be considered modular where each component is capable of binding the Ag regardless of the others. However, some analyses suggest that Ag binding warrants a more integrative view of the relationships between the different components of the Ab.

The binding-sites of interacting proteins are usually composed of surface patches that have good shape and electrostatic complementary (15, 75, 76). It has been shown that CDRs are characterized by an amino-acid composition that is different from that of other protein loops (77) and also from other types of protein–protein interfaces (58). Thus, one would expect that epitopes, just like paratopes, should have a distinct amino-acid composition. However, several recent analyses (51, 53) have shown that this is not the case: while epitopes differ from other types of interfaces (10, 29, 60), their amino-acid composition is virtually the same as that of non-epitopic surface residues.

Several studies have shown that each CDR has its own unique amino-acid composition, different from the composition of the other CDRs (52, 58, 78). Additionally, we have shown that each CDR has a unique set of contact preferences, therefore, favoring certain amino-acids over others (52). Dividing epitope residues into six subsets according to the CDR they bind, we found that each of the subsets has a distinct amino-acid composition, distinguishable from non-epitope surface (52). In other words, when the six subsets of epitope residues are considered together the unique composition of each subset disappears so that the overall amino-acid composition of the entire epitope is indistinguishable from the rest of the surface. Pathogenic epitopes may have evolved to resemble Ag surface to escape recognition. On the other hand, the integration of the six CDRs together, each with its own unique amino-acid composition and contact preferences, could be the evolutionary response of the immune system that enables Abs to recognize virtually any surface patch on the Ag.

Despite this integrated effect of the CDRs, Abs can be also considered as a modular system, composed of different elements (such as the Fab, VH and VL, or the six CDRs), which may bind the Ag on their own. Such smaller Ab fragments that retain Ag binding affinity and specificity, hold a great potential for drug design (79–81) as they have improved pharmacokinetics, tissue and tumor penetration, and can be produced more economically (80, 81). They may also be combined with other fragments to yield better binders. Although such smaller fragments cannot induce effector function such as complement activation (due to the lack of the constant domains), they may neutralize the targeted Ag. Fab and single-chain variable (scFv) fragments usually maintain specific binding to the Ag (82). VH and VL fragments usually show sticky behavior, low solubility, and reduced Ag binding affinity (83–85), although, they sometime retain specificity to the Ag (83, 85–87).

The CDRs may provide additional level of modularity. According to the commonly accepted hotspot hypothesis, the binding energy of two proteins is largely determined by a very small number of critical interface residues (12, 88–90). Thus, one may wonder whether an individual CDR could bind the Ag on its own provided that it harbors hotspots. Several linear peptides containing one or more of the CDRs that retained Ag specificity have been reported (91–98). Although their affinity was usually in the micromolar range, it could be significantly improved by introducing relatively minor modifications (91, 99). However, many attempts to isolate and design such CDR derived peptides failed (100, 101). One possible reason is that a CDR, on its own, may not fold to the same conformation as in the context of the entire Fab, which may be crucial for binding. Cyclizing the CDR by adding Cys residues at its edges was suggested as a solution for this problem (96, 102–104). Another reason might lie in the fact that many attempts for the design of CDR-derived peptides are made based on CDR-H3, as it is considered to be the most important CDR for Ag binding (67, 105–107). However, the median length of ABR-H2 is substantially longer than that of H3, and both typically form the same number of interactions with the Ag (52). In addition, while ABR-H3 was shown to have the highest contribution to Ag binding energy on average (52), there are individual cases in which other CDRs are the dominant ones (52, 102). It is also possible that in some cases the binding depends on specific contacts from residues in different CDRs, which may preclude the design of CDR-derived peptides that maintain specificity. We have shown (102) that CDRs that are able to bind the Ag on their own have unique characteristics and, thus, can be computationally identified given the Ab-Ag complex structure. This may enhance the design of CDR-derived peptides that are not necessarily based on CDR-H3.

## Non-CDR Determinants That have a Role in Ag Binding

### FR residues

Within the variable domain, the CDRs are believed to be responsible for Ag recognition, while the FR residues are considered a scaffold for the CDRs. However, it is now well established that some of the FR residues may play an important role in Ag binding (32, 108). As mentioned above, many such FR residues were identified during the process of Ab humanization by CDR grafting. While grafting only the CDRs usually results in a significant drop or a complete loss of binding, the binding affinity can be retained by back mutating some of the FR residues to the original murine sequence, emphasizing their role in Ag binding (26, 109–115).

Framework region residues that affect Ag binding can be divided into two categories. The first are FR residues that contact the Ag, thus are part of the binding-site (108, 109, 111, 116–123). Some of these residues are close in sequence to the CDRs (in fact they may be within the boundaries of CDRs according to some CDR identification methods, but not according to others, as shown in Figure 3). Other residues are those that are far from the CDRs in sequence, but are in close proximity to it in the 3-D structure. In particular, a loop in the heavy chain FR-3, sometimes referred to as CDR-H4, accounts for 1.3% of human Ab-Ag contacts (78, 124). This CDR-H4 is also enriched (in human Abs) in somatic hypermutations (Burkovitz et al., submitted). Figure 4 shows positions that are not in the CDRs but are in contact with the Ag in many Abs [e.g., the one marked by an orange arrow (4A), which corresponds to CDR-H4].

In the second category of FR residues that affect Ag binding, are residues that are not in contact with the Ag, but affect Ag binding indirectly (108, 109, 120, 121). These residues can be further divided to those that are in spatial proximity to the CDRs, and those that are not. The former are assumed to affect binding by providing a structural support to the CDRs, enabling them to adopt the right conformation and orientation, shaping the binding-site required for Ag binding (32). For example, it has been suggested that a certain position in heavy chain FR-3, close in structure but not in sequence to CDR-H1 and CDR-H2, affects the orientation of CDR-H2 relative to CDR-H1 in such a way that a large side-chain packs between them and separates them while a small side-chain allows them to be closer to each other (109, 120). Nevertheless, this is not always true, as was shown in the case of the anti-lysozyme D1.3 Ab: while mutating Lys in this position to either Val, Ala, or Arg resulted in affinity difference, no structural change was observed (121).

Framework region residues that are more distant from the paratope are suggested to play a role in maintaining the overall structure of the Fv domains (32). However, these FR residues may also affect the Ag binding-site itself, by directing the relative orientation of the VH vs. the VL, and thus the orientation of the CDRs relative to each other (125–128). In particular, FR-2 residues were shown to play an important role in VH-VL interaction (129). Moreover, Masuda et al. (130) pointed to a specific position in the FR-2 loop, which controls the strength of the VH-VL interaction as well as its dependence on Ag binding. We have shown that the conformation of this loop changes upon Ag binding more than other residues in the FRs, and that the binding related conformational changes in this loop are similar in their magnitude to those of the CDRs (107). The potential role of the VH-VL interface in Ag binding is further supported by the observation that residues that are in the VH-VL interface (and are not a part of the Ab-Ag interface), are more likely to be mutated during the somatic hypermutation process, than residues that are not in either of these interfaces (Burkovitz et al., submitted).

Understanding the role of FR residues in Ag binding is crucial for efficient Ab design in general and for humanization in particular. Specifically, knowing in advance which FR residues may affect Ag binding, one may consider back-mutating these residues into their murine sequence, to improve affinity during CDR grafting. To this end, attempts were made to identify positions that contribute to Ag binding in multiple cases (32, 113, 119). For example, Haidar et al. (32) used a non-redundant dataset of Ab-Ag complex structures to identify positions that frequently contact the CDRs, and combined these positions with those that were back-mutated frequently in the humanization literature. The 17 FR positions they identified were successfully used to design a combinatorial library for Ab humanization. Additional Abs, for which structures of both wild-type and a mutant(s) are available, may reveal the structural mechanisms by which each FR position affects Ag binding.

### Constant region

Until recently, Ab constant domains were considered responsible for the isotype and for effector function, such as complement activation, Fc receptor binding, avidity, and serum half-life (131). However, many studies now provide a strong evidence for a role for the constant region in Ag binding (131–147). There are many examples of Abs with identical variable domains but different isotypes that bind the same Ag with a different affinity or specificity (134–146). For instance, two Abs sharing identical variable domains but expressing different isotypes were shown to bind tubulin with significantly different affinities (135). Consistent with these studies, it has been shown that the complex of HEL and the Fv version of the HyHEL-10 Ab has an order of magnitude lower dissociation constant than the complex of HEL with the Fab version of this Ab (147). A probable explanation for this phenomenon would be an allosteric influence of the constant domains on the structure of the variable domains. Indeed, several structural studies provided some evidence for such structural effects (133, 146, 147). For example, Janda et al. (133) analyzed by Circular Dichroism (CD) spectra four different Ab isotypes of the 3E5 family that share identical variable domains, and showed that the different isotypes undergo different structural changes upon binding a common Ag. Similar results were obtained for anti-nuclear Abs as well: Xia et al. (146) compared four different isotypes of the PL9–11 anti-nuclear Ab sharing the same variable region, and found that the changes in secondary structure content (as revealed by CD analysis) as well as the wave length shifts of tryptophan fluorescence emission, upon Ag binding, are both isotype dependent. Recently, Tudor et al. (144) showed that this allosteric effect may control not only Ag binding affinity and specificity, but also the epitope recognized. They showed that two anti-HIV-1 IgG1 and IgA2 Abs with identical variable regions, recognize only partially overlapping epitopes.

Differences in affinity and specificity of Abs with the same variable region but different isotypes may play a role in autoimmunity if they occur in a self-reactive Ag. For example, different isotypes have been shown to be associated with different clinical outcomes for lupus erythematosus: a set of anti-PL9–11 Abs sharing the same variable domain but different isotypes were shown to bind DNA and chromatin, as well as the renal Ags, with different affinities that were associated with significant differences in renal pathogenicity *in vivo* and survival (148).

Several studies have suggested that allosteric effects in Abs may occur on the other direction as well: structural changes in the variable region caused by Ag binding may be transferred into the constant domains, potentially influencing effector activation and cellular response (131, 149–151). For example, Oda et al. (149) showed that the binding of staphylococcal protein A (SPA) or streptococcal protein G (SPG) to the constant region was inhibited by hapten binding in several Abs. A different example was provided by Horgan et al. (151) who observed differences in complement activation of two Abs which differ only in their VH domain.

An allosteric effect in Abs is further supported by a systematic computational analysis we have performed on all available free and Ag-bound pairs of structures (107). Many of the Ag-binding-related structural changes occur distant from the Ag binding-site, including changes in the relative orientation of the heavy and light chains in both the variable and constant domains as well as a change in the elbow angle between the variable and the constant domains. Moreover, the most consistent and substantial conformational change outside of the binding site was found in a loop in the heavy chain constant domain, which is a part of the CH1-CL interface, and is involved in complement binding (152).

What could be the mechanism for these allosteric effects? Changes in the constant domains sequence (different isotypes of the same Ab) or in its conformation (e.g., by effector binding) may lead to a rearrangement of the constant domains relative to each other and relative to the variable domains, which may result in a change to the VH-VL relative orientation (72), thus re-shaping the Ag binding-site (153–155).

The potential influence of the constant region on Ag affinity or specificity suggests that the process of class-switch may be considered, in combination with somatic hypermutations, as a mechanism for Ab diversity (131, 132). Engineering of an Ab of interest is usually associated with the optimization of its affinity to the Ag. Since the constant region may affect this affinity, the isotype selected should be carefully considered. Moreover, the constant region should be taken into account in vaccine design as well since different isotypes may bind the pathogenic Ag with different affinities, thus affecting the response to infection. For example, the anti HIV-1 IgG1 and IgA2 Abs mentioned above share the same variable region, nevertheless, they have been shown to block HIV-1 infection differently (144). While IgA2 blocked HIV-1 transcytosis and CD4^+^ cell infection more efficiently, IgG1 and IgA2 act synergistically to block HIV-1 transfer from Langerhans cells to T cells. Thus, it has been suggested that a mucosal IgA-based vaccine response should complement an IgG-based vaccine response in blocking HIV-1 transmission.

## Concluding Remarks

As Abs are one of the most versatile naturally occurring biosensors, it is of high importance to decipher the structural and molecular mechanisms by which they recognize and bind their Ags. Such knowledge is crucial for understanding immunity, may enable better prediction of Ab epitopes, and assist in Ab engineering.

While the commonly accepted view has been that CDRs hold the key for Ab-Ag recognition, recent findings indicate that not all the positions in the traditionally defined CDRs are important for binding. Furthermore, it has been shown that many positions that contribute critically to the binding energy reside outside of the transitional CDRs. Moreover, different CDR identification methods may often identify radically different stretches as “CDRs,” indicating that CDRs are not well defined and thus are not necessarily a good proxy for the binding site. The hypervariable loops that accommodate the CDRs differ significantly from each other on various aspects. Understanding the way in which their binding preferences are integrated to yield the overall specificity of the Ab is an intriguing structural and biophysical challenge.

Accumulation of recent data suggests that elements that may be spatially distant from the Ag binding site also play a crucial role in Ag recognition. The unorthodox suggestion that non-local and even allosteric effects influence epitope recognition warrants additional analysis and research.

Addressing the open questions regarding the structural basis of Ag recognition requires additional structural data in the form of crystal structures of Abs bound to Ags of different types (proteins, peptides, nucleic acids, and haptens). Large-scale analysis of such structures will allow for the generation and testing of new hypotheses regarding the way in which Abs find and bind their epitopes.

## Conflict of Interest Statement

The authors declare that the research was conducted in the absence of any commercial or financial relationships that could be construed as a potential conflict of interest.
